# Revisiting the Role of Eotaxin-1/CCL11 in Psychiatric Disorders

**DOI:** 10.3389/fpsyt.2018.00241

**Published:** 2018-06-14

**Authors:** Antonio L. Teixeira, Clarissa S. Gama, Natalia P. Rocha, Mauro M. Teixeira

**Affiliations:** ^1^Neuropsychiatry Program & Immuno-Psychiatry Lab, Department of Psychiatry & Behavioral Sciences, McGovern Medical School, University of Texas Health Science Center at Houston, Houston, TX, United States; ^2^Laboratório Interdisciplinar de Investigação Médica, Faculdade de Medicina, Universidade Federal de Minas Gerais, Belo Horizonte, Brazil; ^3^Molecular Psychiatry Laboratory, Hospital de Clínicas de Porto Alegre, Programa de Pós-Graduação em Psiquiatria e Ciências do Comportamento, Universidade Federal do Rio Grande do Sul, Porto Alegre, Brazil

**Keywords:** Eotaxin-1, CCL11, schizophrenia, bipolar disorder, depression, aging, Alzheimer's disease

## Abstract

Eotaxin-1/CCL11 is a chemokine originally implicated in the selective recruitment of eosinophils into inflammatory sites during allergic reactions, being thoroughly investigated in asthma, allergic rhinitis, and other eosinophil-related conditions. Eotaxin-1/CCL11 is also involved with a skewed immune response toward a type-2 (Th2) profile. In addition to its role in immune response, recent studies have shown that eotaxin-1/CCL11 is associated with aging, neurogenesis and neurodegeneration, being able to influence neural progenitor cells, and microglia. Increased circulating levels of eotaxin-1/CCL11 have been described in major psychiatric disorders (schizophrenia, bipolar disorder, major depression), sometimes correlating with the severity of psychopathological and cognitive parameters. As similar findings have been reported in neurodegenerative conditions such as Alzheimer's disease, it has been hypothesized that mechanisms involving eotaxin-1/CCL11 signaling may underlie the “accelerated aging” profile commonly linked to psychiatric disorders. Future studies must determine whether eotaxin-1/CCL11 can be regarded as a prognostic biomarker and/or as therapeutic target for resistant/progressive cases.

## Introduction

There is a robust body of evidence showing altered circulating levels of immune cells and molecules in patients with psychiatric disorders, usually indicating a low-grade systemic inflammation (1, 2). Immune markers have been regarded as potential biomarkers in Psychiatry due to the role played by the immune system in the physiopathology of major psychiatric disorders and the relatively easy access to them (3).

Chemokines—the contraction of “chemotactic” and “cytokine”—constitute a large family of low molecular-weight cytokines whose main action is the recruitment of leukocytes into inflammatory sites (4, 5). Leukocyte recruitment is a highly regulated process, and chemokines are implicated in integrin-mediated adhesion of rolling leukocytes on endothelial cells among other effects. Chemokines are divided into four families based on the relative position of their cysteine residues and their function, being the CCL and CXCL the two largest families. They act by binding to seven-transmembrane G protein-coupled receptors, hence, activating signaling cascades that lead to shape rearrangement and cell movement (4, 5).

Due to the interest in investigating immune biomarkers and their role in the physiopathology of psychiatric disorders, chemokines have been explored in different conditions, including major depression, bipolar disorder, and schizophrenia (6, 7). Our group was one of the very first to systematically evaluate the potential of chemokines as biomarkers of psychiatric disorders. In 2008, we reported increased serum levels of eotaxin-1/CCL11, but not other chemokines, in patients with chronic schizophrenia compared to age and gender-matched controls (8). Subsequent studies extended this finding to propose a role for eotaxin-1/CCL11 as an aging-related biomarker in psychiatry.

In this non-systematic mini-review we revisit the actions originally and currently ascribed to eotaxin-1/CCL11, highlighting the emerging role of eotaxin-1/CCL11 in psychiatric disorders, mainly schizophrenia and mood disorders.

## Eotaxin-1/CCL11: from eosinophil recruitment to a broader pathophysiological role

In 1994, studying a model of allergic inflammation, the group of Prof. Timothy Williams at the National Heart and Lung Institute, London, described a new protein capable of selectively recruiting eosinophils, but not neutrophils, into inflammatory sites. The protein named “eotaxin” was a potent stimulator of both rodent and human eosinophils *in vitro* (9, 10). Subsequent studies confirmed the role of “eotaxin” as a potent eosinophil chemoattractant cytokine, also describing its main receptor, the CC chemokine receptor 3 (CCR3) (11–13). “Eotaxin” was renamed eotaxin-1 after eotaxin-2 and eotaxin-3 were identified, and later CCL11 (14). Eotaxin-1/CCL11 can also bind to the CCR2 and CCR4 receptors, but its selectivity to CCR3 is much higher than to the other receptors (15).

**Figure 1:**
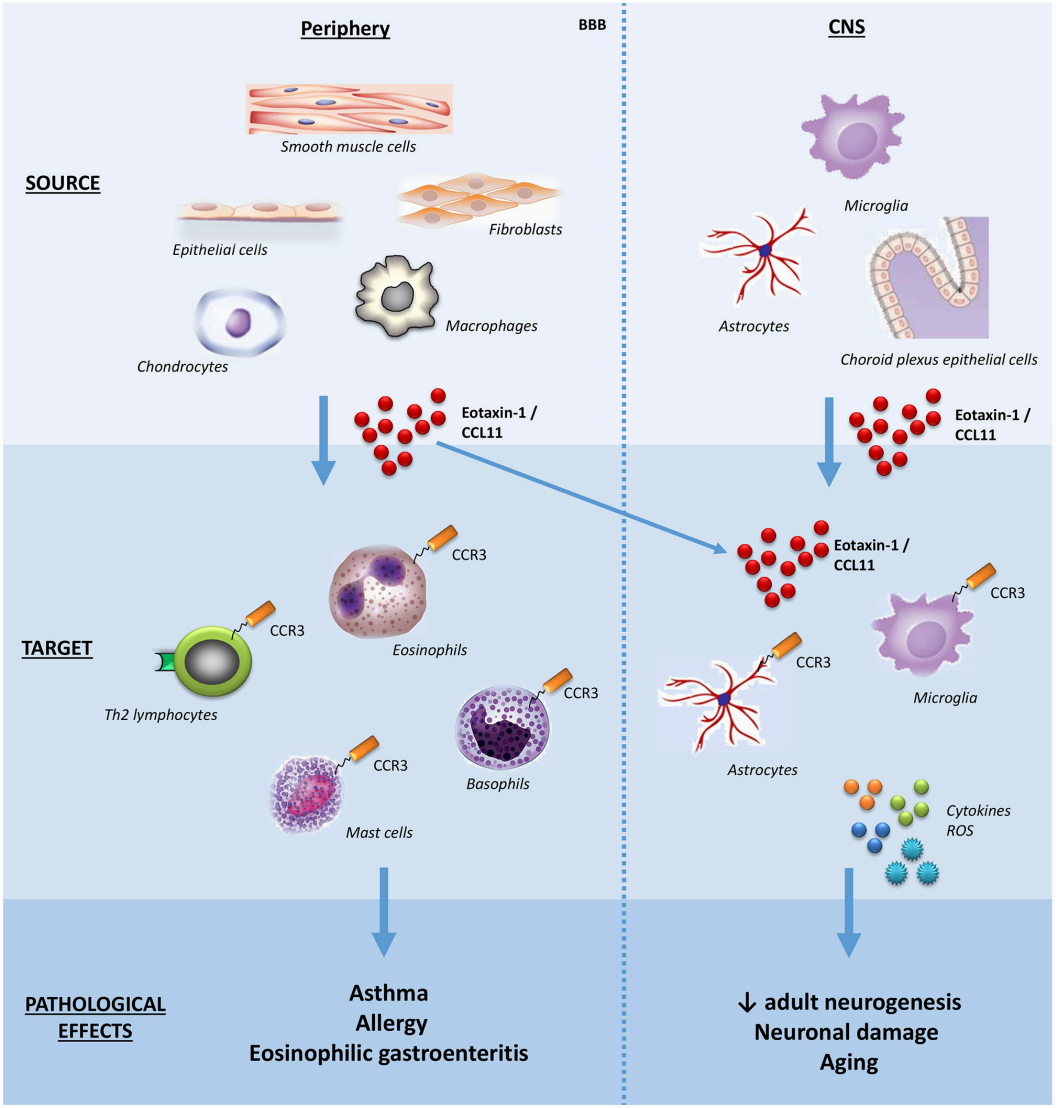
Schematic effects of eotaxin-1/CCL11 in adult subjects. The main sources, targets, and effects of the chemokine eotaxin-1/CCL11 are shown both in peripheral tissues and CNS. Note that eotaxin-1/CCL11 is capable of crossing the BBB and influencing CNS cells. BBB, blood-brain barrier; CNS, central nervous system; ROS, reactive oxygen species.

Eosinophils have been implicated in a broad range of conditions, notably allergic (asthma, rhinitis, and atopic dermatitis) and inflammatory diseases characterized by eosinophil accumulation in tissues (eosinophilic esophagitis, gastroenteritis, and pneumonia), and helminthic diseases (for example, schistosomiasis). Due to the pathological role of eosinophils in asthma and atopic dermatitis, the first studies evaluated the cellular sources of eotaxin-1/CCL11 in the lung and the skin, reporting that epithelial cells, fibroblasts, smooth muscle cells can produce it. Subsequently, other sources of eotaxin-1/CCL11 were reported, including astrocytes, chondrocytes, and tissue resident macrophages. In the central nervous system (CNS), choroid plexus epithelial cells, pericytes, astrocytes, and microglia seem to produce eotaxin-1/CCL11 under inflammatory stimuli (16) (Figure 1).

Once binding to CCR3 receptors expressed on the cell surface of eosinophils, eotaxin-1/CCL11 activates a series of intracellular signaling cascades, leading to eosinophil recruitment to inflammatory sites. Eosinophils are source of cytotoxic granular proteins and growth factors responsible, respectively, for tissue damaging and remodeling implicated in the physiopathology of several diseases such as asthma. Therefore, the selective blockade of the CCR3-eotaxin-1/CCL11 axis could impair eosinophil recruitment, representing an attractive target for the treatment of asthma, allergic rhinitis, and other eosinophil-related conditions (17). Indeed, there have been early phase clinical trials with CCR3 antagonists for asthma and, more recently, a therapeutic antibody against eotaxin-1/CCL11 (Bertilimumab) for allergic rhinitis (10, 18). From a clinical perspective, eotaxin-1/CCL11 has also been evaluated as a biomarker of human diseases (19). A systematic review of the literature involving 30 studies showed that blood and sputum eotaxin-1/CCL11 concentrations were consistently elevated in patients with asthma, being negatively correlated with lung function, indicating the potential use of eotaxin-1/CCL11 as a biomarker for the diagnosis and assessment of asthma severity and control (20).

Besides eosinophils, the chemokine receptor CCR3 is expressed on basophils, mast cells and Th2 lymphocytes, the latter involved with the production of the so-called Th2 cytokines (interleukins, IL; IL-4, IL-5, IL-13) (Figure 1). Accordingly, eotaxin-1/CCL11 has been implicated in skewing the immune response toward a type-2 (Th2) response (21).

In addition to immunomodulation, other effects of eotaxin-1/CCL11 have been described. For the current discussion, it is worth emphasizing its effects on the CNS, and mentioning that eotaxin-1/CCL11 can cross the unaltered blood-brain barrier (22). Krathwohl and Kaiser (23) showed that eotaxin-1/CCL11 reversibly inhibits neural progenitor cell proliferation *in vitro* in isolated cells, neurospheres, and in hippocampal slice cultures without affecting their ability to form both neurons and astrocytes (23). In an elegant study using parabiosis, (24) showed that plasma of aging mice or eotaxin-1/CCL11 administration to young mice decreased adult neurogenesis and impaired memory and learning, proposing a major role for this chemokine in the age-related decline of hippocampal function (24). Later, it was demonstrated that while there was no direct effect of eotaxin-1/CCL11 on neurons, this chemokine was able to promote microglia migration and activation with subsequent production of reactive oxygen species, potentiating glutamate-induced neuronal death (25). In this same direction, our group described elevated levels of eotaxin-1/CCL11 in the hippocampus along with impaired neurogenesis and cognitive/memory impairment in a mouse model of cerebral malaria (26). Altogether, these findings suggest a link between eotaxin-1/CCL11 and aging, neurogenesis impairment and neurodegeneration.

## Studying eotaxin-1/CCL11 in psychiatric disorders

To the best of our knowledge, the first studies assessing eotaxin-1/CCL11 in psychiatric disorders were published in 2008 (8, 27) (Table 1).

**Table 1:** Main findings regarding eotaxin-1/CCL11 levels in major psychiatric disorders.

**Psychiatric disorder**	**Findings**
Schizophrenia	Increased blood levels; Negative correlation with telomere length and gray matter volume; Negative correlation with cognitive measures; Positive correlation with negative symptoms.
Bipolar disorder	Increased blood levels; Association with illness stage.
Major depression	Increased blood levels; Association with suicidal ideation.
Dysthymia	Increased blood levels.
Obsessive-compulsive disorder	Blood levels similar to controls.
Autism spectrum disorder	Increased blood levels.
Substance abuse disorder	In heroin dependent subjects, increased blood levels and association with age. In alcohol dependent subjects, decreased blood levels, especially in women and with comorbid psychiatric disorders.

Simon et al. (27) simultaneously assessed the serum levels of 22 cytokines/chemokines, including eotaxin-1/CCL11, in 49 patients with major depression and 49 matched controls, reporting increased levels of the molecule in a context of “generalized chronic inflammatory state” (27). Later we found similar results in an independent cohort of patients with major depression, indicating that increased serum levels of eotaxin-1/CCL11 were particularly associated with suicidal ideation (28). Nevertheless, a recent systematic-review and meta-analysis of studies evaluating eotaxin-1/CCL11 in depression (not necessarily major depression) including 454 participants (230 cases vs. 224 controls) failed to identify significant difference between CCL11 measurements in depressed and control subjects (29). The fact that this meta-analysis also included studies with subjects presenting with medical comorbidities (including inflammatory-related conditions) and possibly milder forms of depression may explain the discordance with the first reports.

Teixeira et al. (8) evaluated the serum levels of six chemokines (CCL2, CCL3, CCL11, CXCL8, CXCL9, CXCL10) in 40 patients with chronic schizophrenia and 20 controls. Only the levels of eotaxin-1/CCL11 were increased in the patients compared to controls, but no association was found between chemokine levels and clinical parameters such as severity of positive and negative symptoms, and involuntary movements (8). Soon after, we evaluated the serum levels of a set of chemokines (CCL2, CCL3, CCL11, CCL24, CXCL8, CXCL9, CXCL10) in 30 euthymic patients with bipolar disorder and 30 matched controls (30). Patients with bipolar disorder showed increased levels of IP-10/CXCL10, lower levels of eotaxin-2/CCL24 and similar levels of the other chemokines compared to controls. Taking into account that IP-10/CXCL10 is associated with a Th1 response, and eotaxin-2/CCL24 (as eotaxin-1/CCL11) is related to a Th2 response, this result suggested an imbalance of Th1/Th2 cytokines toward a Th1 profile in bipolar disorder (30). Based on the chemokine studies, at this point we were very excited with the hypothesis that schizophrenia would be associated with a preferential activation of Th2 lymphocytes as previously proposed by Muller et al. (31), while bipolar disorder with the activation of Th1 lymphocytes.

Nevertheless, subsequent studies failed to confirm immune response polarization in schizophrenia or bipolar disorder (1). Studying the plasma levels of six chemokines (CCL2, CCL3, CCL11, CCL24, CXCL8, and CXCL10) in an independent sample composed of 70 bipolar disorder type I patients (35 in euthymia and 35 in mania) and 50 matched controls, we found increased levels of IP-10/CXCL10 and eotaxin-1/CCL11 in patients regardless of the mood phase (32). Magalhaes et al. (33) also reported increased levels of eotaxin-1/CCL11 in patients with bipolar disorder recruited from the community (33). Actually, there are similarities in the pattern of cytokine changes in schizophrenia and bipolar disorder during acute and chronic phases of the respective illness, possibly indicating shared pathophysiological pathways leading to immune dysfunction (34). Different results were obtained when evaluating patients with obsessive-compulsive disorder.

More recently, we showed that late-stage patients with bipolar disorder, defined by a clinical staging model taking into consideration the number of previous mood episodes, comorbidities, and cognitive and social functioning, tended to express higher serum levels of eotaxin-1/CCL11 than early-stage patients and controls (35). This study supported the findings of altered levels of eotaxin-1/CCL11 in bipolar disorder, and indicated an increase in the circulating levels of this chemokine with progressive clinical deterioration observed in this condition. Moreover, taking into account the evidence implicating eotaxin-1/CCL11 in the age-related decline of hippocampal function, including memory and learning impairment (24, 25), it corroborates the hypothesis of “accelerated aging” in bipolar disorder (36). We observed similar findings in schizophrenia (37) as patients with chronic illness (>20 years of diagnosis) had higher circulating levels of eotaxin-1/CCL11 than age-matched controls, while patients with early illness (< 5 years of diagnosis) did not differ from their age-matched controls.

In a recent study comprising 48 patients with schizophrenia and 64 controls, we had the chance to reiterate the hypothesis of “accelerated aging” in this major psychiatric condition (38). In comparison with controls, patients had decreased telomere length (a biological marker of aging) and gray matter volume (a neuroimaging marker of aging/degeneration), increased eotaxin-1/CCL11 levels, and worse memory performance as assessed by the Hopkins Verbal Learning Test. More importantly, shorter telomere length was related to increased levels of eotaxin-1/CCL11, and both biomarkers were related to reduced gray matter volume, all of which were related to worse memory functioning. Further supporting a role for eotaxin-1/CCL11 in human cognition, (39) reported increased levels of this chemokine in patients with schizophrenia compared to controls, and a negative correlation with working memory (Visual Working Memory Test) and a positive correlation with cognitive flexibility (Plus-Minus Task) (39). Noto et al. (40) also reported a positive correlation between the severity of negative symptoms (i.e., apathy, blunted affect, poverty of speech, social withdrawal) and eotaxin-1/CCL11 levels (40). An independent group corroborated these results, reporting positive correlation between eotaxin-1/CCL11 levels with age, duration of schizophrenia, and severity of negative symptoms (41). Although correlational, these findings suggest that eotaxin-1/CCL11 may influence the function of different neural circuits, including dorsolateral, and ventromedial fronto-striatal circuits.

In line with the indirect findings implicating eotaxin-1/CCL11 in “accelerated aging” in bipolar disorder and schizophrenia, elevated plasma levels of eotaxin-1/CCL11 have been observed in neurodegenerative diseases, mainly Alzheimer's disease (42, 43). It remains to be established whether these levels correlate with the rate of disease/neurodegeneration progression.

Finally, it is worth mentioning that altered levels of eotaxin-1/CCL11 has been associated with children and adolescent psychopathology, including autism spectrum disorder (44, 45), and other psychiatric conditions, including dysthymia (46), obsessive-compulsive disorder (47), and substance use disorders (48, 49).

## Concluding remarks

Eotaxin-1/CCL11 has been associated with major psychiatric disorders. This finding undermines its role as a diagnostic marker, but suggests that this chemokine may be involved in shared pathophysiological mechanisms among them, especially those implicated in “accelerated aging.” In this regard, eotaxin-1/CCL11 seems very promising as it has been associated with markers of aging and degeneration; also correlating with cognitive measures. There are several opportunities here such as: (i) longitudinal studies with careful psychopathological and cognitive phenotyping aiming to determine its prognostic value; (ii) neuroimaging studies to evaluate its association with neurodegenerative changes (e.g. PET analysis of beta-amyloid and tau burden). For example, eotaxin-1/CCL11 has been used as a biomarker in clinical trials in asthma (50, 51).

In sum, although preliminary, there is evidence supporting that eotaxin-1/CCL11 may exert physiological and pathological effects in the CNS. If confirmed these pathological effects, it is tempting to propose strategies against eotaxin-1/CCL11 or its CCR3 receptor for the treatment of severe, progressing, and/or refractory cases of major psychiatric disorders. There have been clinical trials with CCR3 antagonists and anti-eotaxin-1/CCL11 neutralizing antibodies in inflammatory human diseases with encouraging results.

## Author contributions

AT and MT conceived the original idea. AT and CG performed the literature review and critically analyzed the data. AT wrote the first draft of the manuscript with inputs from CG and NR. MT critically reviewed the manuscript.

## Conflict of interest statement

The authors declare that the research was conducted in the absence of any commercial or financial relationships that could be construed as a potential conflict of interest.
